# A Framework for Measuring the Progress in Exoskeleton Skills in People with Complete Spinal Cord Injury

**DOI:** 10.3389/fnins.2017.00699

**Published:** 2017-12-12

**Authors:** Rosanne B. van Dijsseldonk, Hennie Rijken, Ilse J. W. van Nes, Henk van de Meent, Noel L. W. Keijsers

**Affiliations:** ^1^Department of Research, Sint Maartenskliniek Research, Nijmegen, Netherlands; ^2^Department of Rehabilitation, Sint Maartenskliniek, Nijmegen, Netherlands; ^3^Department of Rehabilitation, Radboudumc, Nijmegen, Netherlands

**Keywords:** spinal cord injury, exoskeleton, paraplegia, ambulation, skills

## Abstract

For safe application of exoskeletons in people with spinal cord injury at home or in the community, it is required to have completed an exoskeleton training in which users learn to perform basic and advanced skills. So far, a framework to test exoskeleton skills is lacking. The aim of this study was to develop and test the hierarchy and reliability of a framework for measuring the progress in the ability to perform basic and advanced skills. Twelve participants with paraplegia were given twenty-four training sessions in 8 weeks with the Rewalk-exoskeleton. During the 2nd, 4th, and 6th training week the Intermediate-skills-test was performed consisting of 27 skills, measured in an hierarchical order of difficulty, until two skills were not achieved. When participants could walk independently, the Final-skills-test, consisting of 20 skills, was performed in the last training session. Each skill was performed at least two times with a maximum of three attempts. As a reliability measure the consistency was used, which was the number of skills performed the same in the first two attempts relative to the total number. Ten participants completed the training program. Their number of achieved intermediate skills was significantly different between the measurements X_F_^2^(2) = 12.36, *p* = 0.001. *Post-hoc* analysis revealed a significant increase in the median achieved intermediate skills from 4 [1–7] at the first to 10.5 [5–26] at the third Intermediate-skills-test. The rate of participants who achieved the intermediate skills decreased and the coefficient of reproducibility was 0.98. Eight participants met the criteria to perform the Final-skills-test. Their median number of successfully performed final skills was 16.5 [13–20] and 17 [14–19] skills in the first and second time. The overall consistency of >70% was achieved in the Intermediate-skills-test (73%) and the Final-skills-test (81%). Eight out of twelve participants experienced skin damage during the training, in four participants this resulted in missed training sessions. The framework proposed in this study measured the progress in performing basic and advanced exoskeleton skills during a training program. The hierarchical ordered skills-test could discriminate across participants' skill-level and the overall consistency was considered acceptable.

## Introduction

Worldwide the incidence of Spinal Cord Injury (SCI) is 180,000 cases per annum (Lee et al., 2014) of whom 50% have a complete lesion and become wheelchair-designated for their mobility (Wyndaele and Wyndaele, 2006). A lifetime of sitting has been associated with an increased risk of multiple secondary complications, such as pressure ulcers, spasticity, and worsening of bladder and bowel dysfunction (Jensen et al., 2013; Adriaansen et al., 2016). Exoskeletons (external active orthosis) make it possible for people with paraplegia to regain their standing and walking mobility by generating the basic motions for ambulation e.g., standing-up, sitting-down, standing, and walking. Similar to other standing and robotic gait training devices (Middleton et al., 1997; Dunn et al., 1998; Mirbagheri et al., 2015), exoskeletons have the potential to prevent secondary health complication (Miller et al., 2016). The main benefit of exoskeletons compared to other robotic gait training devices (such as Lokomat®) is that exoskeletons can be used at home and in the community outside of a clinical setting (Federici et al., 2015). However, several risks are identified with exoskeleton use such as falls, joint misalignment, skin damage, software malfunctions, electrical and fire hazard, and user errors (He et al., 2017). So far, the chance and extent of the risks are not well understood (He et al., 2017). Furthermore, manufacturers require an intensive training period before home and community use is allowed.

A prerequisite for safe and independent home and community exoskeleton use, is that users are able to perform basic and advanced exoskeleton skills. Previous research mainly focussed on the basic skills (sit-to-stand, stand-to-sit, and walking) and has shown that basic skills can be learned in a 25 sessions-training program with varying levels of assistance (Spungen et al., 2013; Kozlowski et al., 2015; Platz et al., 2016). The basic skills are highly relevant for use in a clinical setting, but for safe independent community use more advanced skills are required, including arresting gait on command, passing door thresholds, low curbs and ramps and controlling the input device (Spungen et al., 2013; Yang et al., 2015). The control of and interaction with the exoskeleton is diverse across the different exoskeletons available on the market. Moreover, some exoskeletons are more difficult or impossible to control dependent on the level and severity of the SCI of the user (Bryce et al., 2015). Several studies tested advanced skills in a limited number of motor complete SCI patients (Spungen et al., 2013; Hartigan et al., 2015; Kozlowski et al., 2015; Benson et al., 2016; Platz et al., 2016). However, the advanced skills were not tested in a systematic way and for example Spungen et al. concluded that the skills could have been introduced earlier in the training program (Spungen et al., 2013). So far, a systematic framework to structure, test and evaluate exoskeleton skills during a training program is lacking. Therefore, the aim of this study is to develop a framework to measure the progress in the ability to perform exoskeleton skills. The proposed framework consists of exoskeleton skills arranged into a hierarchy so that the difficulty increased with each tested skill. If the exoskeleton skills formed a true hierarchy and a skill was not achieved, it can be assumed that the participant would not achieve all higher skills and would achieve all lower skills. Therefore, arranging the skills into a hierarchy would reduce the time and effort of the exoskeleton-skills-test (Tyson and DeSouza, 2004). Furthermore, it is essential that the exoskeleton-skill-tests in the framework are reliable. Accordingly, the skills had to be performed consistent to reduce the change of misjudging the participants' skill-level.

Before an advanced exoskeleton skill-level can be achieved, an intensive training program with multiple training sessions per week over a longer period of time is required. The risk factors associated with such an intensive training program are still not well understood (He et al., 2017). However, it can be expected that such an intensive training program decreases the risk of falls, joint misalignment and user errors and increases the safety of exoskeleton home and community use. On the contrary, intensive exoskeleton use increases the risk of skin damage and bruises. Previous research concluded that in hospital training with an exoskeleton was safe (Zeilig et al., 2012; Esquenazi, 2013; Kolakowsky-Hayner et al., 2013; Kubota et al., 2013; Spungen et al., 2013). However, other studies disclosed mild to moderate skin damages in half of the participants (five out of ten) (Benson et al., 2016) (four out of seven) (Platz et al., 2016). Other reported complications were a fracture of the talus (Louie et al., 2015) and venous-lymphatic stasis in the lower limbs (Onose et al., 2016). Hence, assessing the occurrence of complications such as skin damage, muscle or joint pain, incontinence problems, device related errors, fractures, venous-lymphatic stasis, and falls during an exoskeleton training program is important for clinical recommendations.

In conclusion, the main objective of this study was to develop and test a framework for measuring the progress to perform basic and advanced exoskeleton skills in a group of individuals with motor complete SCI. The hierarchy and the reliability of the exoskeleton-skills-test in the framework was evaluated. As a secondary outcome, complications such as skin damage, muscle or joint pain, and incontinence problems resulting from the intensive exoskeleton training program were assessed.

## Materials and methods

### Participants

People with paraplegia who gained knowledge about the exoskeleton technology throughout the media and who were interested in testing the potential of an exoskeleton contacted the rehabilitation center of the Sint Maartenskliniek to participate in this study. Eligible persons were adult patients in the chronic phase (>6 months) with a motor complete SCI [American Spinal Injury Association Impairment Scale (AIS) A or B] between Thoracic 1 (Th1) and Lumbar 1 (L1). The exclusion criteria were physical factors that hamper proper functioning of the exoskeleton, such as severe spasticity (Modified Ashworth Scale > 3), taller than 1.90 m or smaller than 1.60 m, bodyweight above 100 kg, and restricted range of motion in the hip, knee, or ankle joint. Other exclusion criteria were inability to control crutches, unable to make a transfer from a chair to a wheelchair without the use of external support, and patients with conditions that could interfere with the motor learning process (e.g., stroke). Potential subjects with an increased risk of adverse events such as patients with osteoporosis, fractures of the lower extremities in the last 2 years, balance disorders, neurogenic heterotopic ossification and pregnancy were also excluded. All participants gave written informed consent in accordance with the Declaration of Helsinki. The study was approved by the medical ethics committee of Arnhem-Nijmegen (2016-2418) and the internal review committee of the Sint Maartenskliniek.

### Procedure

All exoskeleton training sessions and measurements were performed in the sports hall at the rehabilitation center. Prior to the start of the training a brief physical examination by a rehabilitation physician was performed, in which the in- and exclusion criteria of the study were checked. Participants were given twenty-four training sessions of 1.5-h over an 8 week period. Three physical therapists were trained by ReWalk™ Robotics to give the exoskeleton training. During each session at least two physical therapists were present to assure safety. The exoskeleton and the Lofstrand crutches were adjusted to the patients' body composition during the first training session. After each training the physical therapists notated the skills that were practiced. Participants kept a logbook during the entire study including any complications such as skin abrasions, muscle or joint pain, falls, and incontinence problems. The logbook was filled out at least three times a week. To assess the progress in achieved skills the participants' skill-level was tested every 2 weeks during a training session. In total the skill-level was assessed four times during the study, three times with the Intermediate-skills-test and one time with the Final-skills-test.

### Intermediate- and final-skills-test

The Intermediate-skills-test was performed in training week 2, 4, and 6. The Intermediate-skills-test consisted of 27 skills, which were measured separately of each other and were arranged into a hierarchy so that the difficulty increased with each skill. The intermediate skills were sorted into three categories; standing, walking, and advanced skills. Each subsequent category required more control of the user over the exoskeleton. Within each category the complexity of the skills also increased. In the standing skills the feet of the user remained roughly at the same place and participants learned to use their crutches, whereas there was displacement of the feet in the walking and advanced skills. In the walking skills, the increase of difficulty was related to the decrease in level of assistance and number of involuntary stops. In the advanced skills an additional task was performed while walking. The complexity of the task increased from walking turns with a decrease in number of involuntary stops to passing obstacles to passing obstacles that require raising or lowering of the center of mass (walk up and down a martial arts mat). An overview of the 27 skills of the Intermediate-skills-test is given in Table 1. Each intermediate skill was performed at least two times with a maximum of three attempts. An intermediate skill was considered achieved when the skill was performed independent without assistance of the exoskeleton trainer in at least two out of three attempts. The Intermediate-skills-test continued until two skills were not achieved. Participants were allowed to take rest between the various skills tested. A more detailed description of the Intermediate-skills-test is provided in Supplementary Table 1 (available online).

**Table 1:** Assessed exoskeleton skills in the Intermediate-skills-test.

**Category**	**Order**	**Intermediate skill**
Standing skills	1	Weight shifting forward and backward and to the right and left
	2	Touching the wristband during standing
	3	Sit-to-stand
	4	Stand-to-sit
Walking skills	5	Walk 10 m with assistance (with max. 2 stops)
	6	Stop with the preferred leg
	7	Stop with the not preferred leg
	8	Walk 10 m without assistance (with max. 2 stops)
	9	Walk 10 m without assistance (without stops)
Advanced skills	10	Arrest gait at command
	11	Walk a 90° curve to the right (with max. 1 stop)
	12	Walk a 90° curve to the right (without stops)
	13	Walk a 90° curve to the left (with max. 1 stop)
	14	Walk a 90° curve to the left (without stops)
	15	Walk a 180° curve (radius 1.8 m) to the right (with max. 1 stop)
	16	Walk a 180° curve (radius 1.8 m) to the right (without stops)
	17	Walk a 180° curve (radius 1.8 m) to the left (with max. 1 stop)
	18	Walk a 180° curve (radius 1.8 m) to the left (without stops)
	19	Arrest gait nearby a vaulting box (height 1.1 m) and move a cone at chest height
	20	Pass a narrow passage (width 0.8 m) (with max. 1 stop)
	21	Arrest gait nearby a door (width 0.8 m), open the door away from you and enter (with max. 1 stop)
	22	Arrest gait nearby a door (width 0.8 m), open the door toward you and enter (with max. 1 stop)
	23	Arrest gait near a chair (height 0.5 m) and pivot turn to sit down
	24	Pass an upward and downward sloping doorstep (angle up 11.3°and down 16.7°, height 0.03 m) (with max. 1 stop)
	25	Walk up a martial arts mat (height 0.04 m) (with max. 1 stop)
	26	Walk down a martial arts mat (height 0.04 m) (with max. 1 stop)
	27	Walk a slalom around 4 badminton poles (distance between poles 3.0 m) (with max. 2 stops)

**Figure 1:**
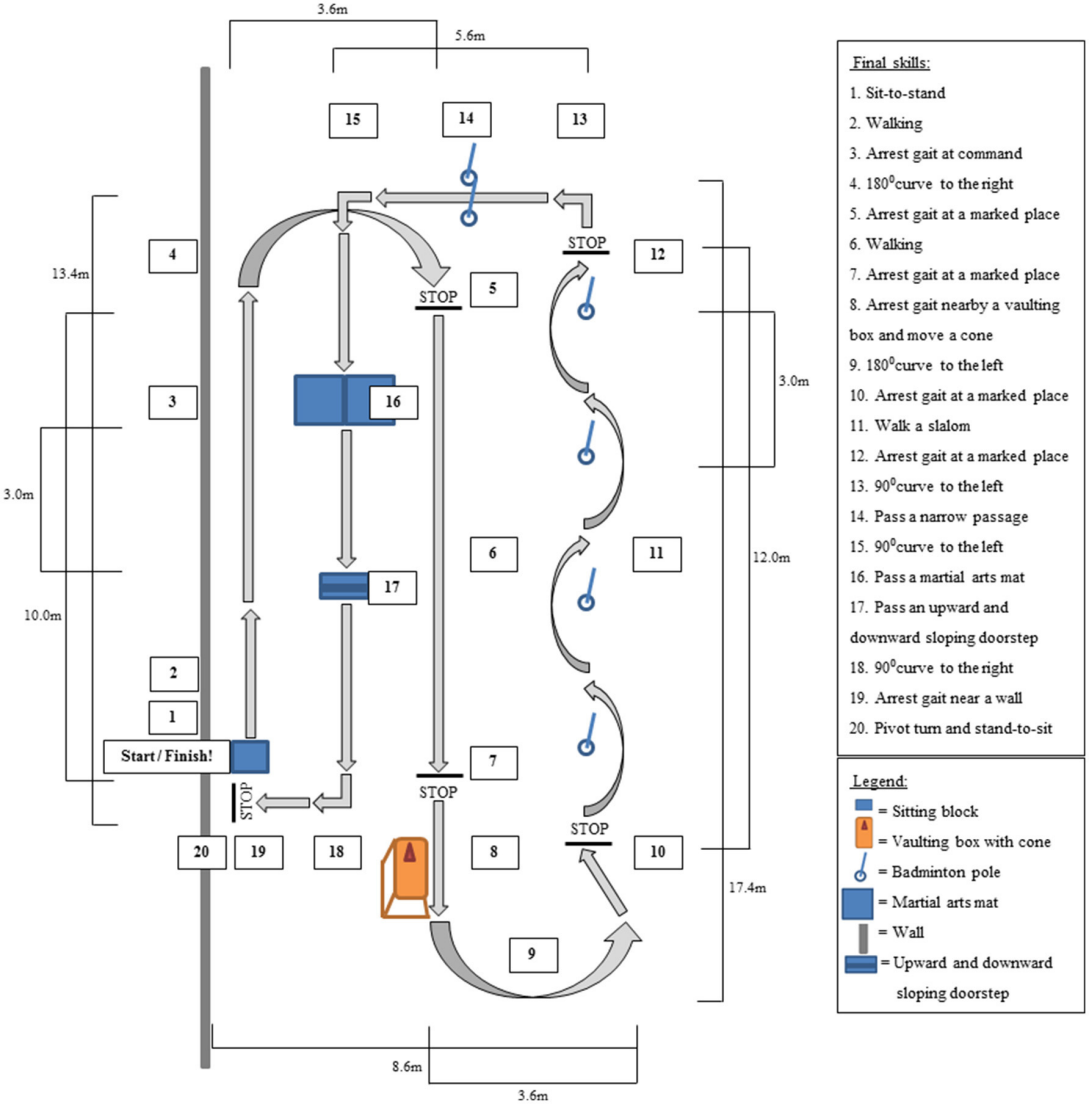
Schematic representation of the top view of the Final-skills-test. Arrows represent the walking direction.

The Final-skills-test was performed during the last training week (week 8) in the final training session. A prerequisite for performing the Final-skills-test was that participants could control the remote control and walk without assistance of the exoskeleton trainer. The Final-skills-test consisted of a fixed set of 20 skills and was performed two times with a 5 min break in between. In contrast to the Intermediate-skills-test, the tested exoskeleton skills were measured in sequence during the Final-skills-test, simulating daily life situations in which skills are rarely performed independent of each other. Moreover, performing skills in sequence made it more difficult to achieve a skill than performing skills independent from each other (e.g., arresting gait immediately after a sharp curve compared to arresting gait independent of the previous action). In the Final-skills-test the focus was on independent performance of skills and the number of stops was not taken into account. Furthermore, the basic intermediate skills (e.g., weight shifting, touching the wristband, sit-to-stand, and assisted walking) are required in performing most of the skills and were not tested separately. In order to assess the test in a sports hall with as little material as possible, the advanced intermediate skill of opening a door was not part of the Final-skills-test. To assure safety, the exoskeleton trainer walked behind the participant but did not intervene unless the participants lost their balance and could fall. The final skills were considered achieved when the participants performed the skills without assistance of the exoskeleton trainer. In Figure 1 a schematic representation of the Final-skills-test is given.

### Equipment

In this study, two wearable robotic exoskeletons that enable powered hip and knee motion from ReWalk™ Robotics were used; (1) the ReWalk™ Rehabilitation System and (2) the ReWalk™ Personal 6.0. The exoskeleton systems provided user-initiated mobility through the integration of a wearable brace support, a computer-based control system and motion sensors. The exoskeleton systems have the Class II FDA clearance for both use in a rehabilitation setting as well as personal use. All participants started training with the ReWalk™ Rehabilitation System. Only participants who met de criteria to perform the Final-skills-test used the ReWalk™ Personal 6.0 system as well.

### Data and statistical analysis

To assess if the proposed framework measured the ability to perform basic and advanced exoskeleton skills throughout an exoskeleton program, the skill-tests in the framework should measure progression in the number of achieved skills and show distinct skill-levels between participants. The skills should be arranged into a hierarchical order of difficulty. Moreover, the skills tested in the framework should be performed consistent. In addition, the relation between the Intermediate- and Final-skills-test was determined.

### Achieved intermediate skills

The number of achieved skills was analyzed using descriptive statistics (median and ranges). Differences in the number of achieved skills between the three Intermediate-skills-test was assessed with the non-parametric Friedman test (α = 0.05). In case of a significant Friedman test, Wilcoxon *post-hoc* test with Bonferroni correction (α = 0.017) was used to determine changes. The number of participants who showed the expected increase in number of achieved skills over the three intermediate measurements was determined. Each intermediate skill was also analyzed separately for the number of times a skill was achieved.

#### Hierarchy of the skills

The hierarchy of the skills tested in the Intermediate-skills-test was analyzed according to two measurements (1) the rate of participants achieving each intermediate skill and (2) the coefficient of reproducibility (Tyson and DeSouza, 2004). Both tests are based on the theoretical expectation that the participants ability to achieve a skill would decrease as the difficulty of the task increased. For a more detailed description see Tyson and DeSouza (2004). The coefficient of reproducibility was calculated with the formula described by Tyson and DeSouza: Coefficient of reproducibility = 1 − scaling errors/(number of skills × number of observations; Tyson and DeSouza, 2004). In which scaling errors is the number of participants who did not achieve the skills in the predetermined order. Since participants progressed during the training each intermediate skills measurement was considered as a separate observation in the analysis. A coefficient of reproducibility of at least 0.9 was considered acceptable (Guttman, 1944; Tyson and DeSouza, 2004).

#### Achieved final skills

The number of achieved final skills was analyzed using descriptive statistics (median and ranges). Each final skill was also analyzed separately for the number of times a skill was achieved. The correlation between the number of achieved skills in each skills-test was assessed with Kendall's rank correlation coefficient (Kendall's Tau).

#### Consistency

The consistency in the number of exoskeleton skills which were performed the same in the first two attempts (successful-successful or failure-failure) relative to the total number of performed skills was used as a reliability measure of the Intermediate-skills-test and the Final-skills-test. An overall consistency of >70% was considered reliable. Each intermediate skill and final skill was also analyzed separately for the number of times a skill was tested, performed consistent and performed successful.

#### Complications

To assess the occurrence of complications during an exoskeleton training program both the physical therapists and participants filled out a logbook after each training session including any complications. The reported complications such as the number of skin damages, location of skin damages, incidence of reported muscle or joint pain, number of incontinence problems, device related errors, fractures, venous-lymphatic stasis and falls during the exoskeleton training program were analyzed using descriptive statistics.

## Results

### Participants

Out of 12 participants 10 (83%) completed the training program. Reasons for not completing the training program were inability to learn the basic skills of the exoskeleton (stopped after seven training sessions and performed the first Intermediate-skills-test) and absence of perceived benefit (stopped after two training sessions and did not perform an Intermediate-skills-test). Eleven participants completed at least one Intermediate-skills-test, the data of these participants was used in the analysis of the hierarchy and consistency of the intermediate skills. Due to time constraints, one participant was not able to perform the skills a second time during the Final-skills-test. For this participant the set of final skills was repeated twice one week later. The data of the second Final-skills-test was only used for the consistency analysis whereas the first Final-skills-test was used for the analysis of the achieved skills after the training program. We do not expect that this had an impact on the outcome of the current study. An overview of the patient characteristics is given in Table 2.

**Table 2:** Patient characteristics.

	**Total (*N* = 12)**
Gender (male/female)	7/5
Age (years), median [range]	42 [24–56]
Level of SCI, median [range]	Thoracic 9 [4–11]
Post-injury (months), median [range]	75 [24–276]
AIS^*^ (A/B)	11/1

* *AIS, American Spinal Injury Association Impairment Scale*.

### Achieved intermediate skills

**Figure 2:**
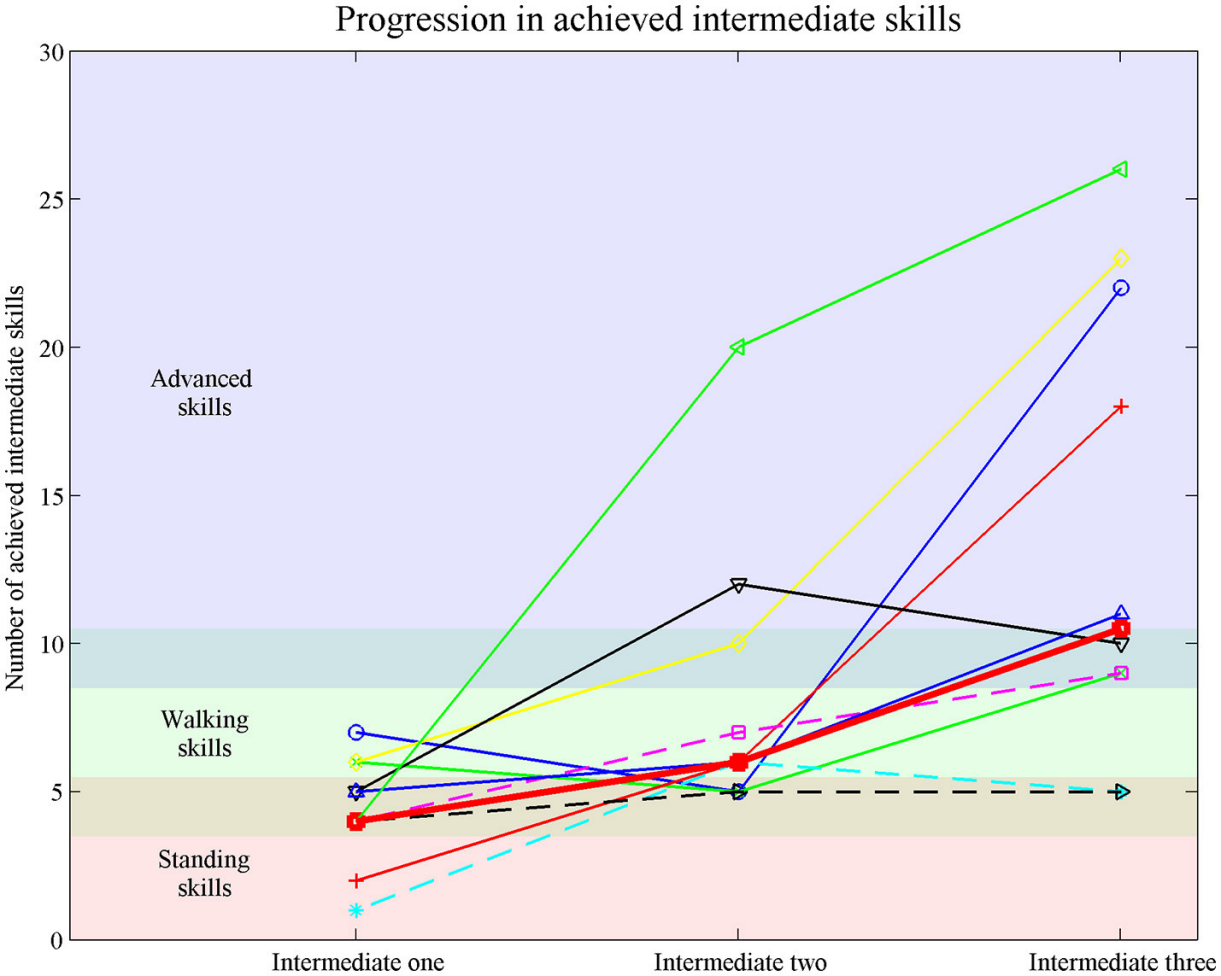
Achieved intermediate skills measured with the Intermediate-skills-test one, two and three. Each line represents a participants. Thick red line represents the median achieved intermediate skills. Dotted lines represent participants who did not perform the Final-skills-test.

The Friedman test revealed a significant difference between the number of achieved intermediate skills between the measurements [X_F_^2^(2) = 12.36, *p* = 0.001]. *Post-hoc* analysis revealed that the achieved intermediate skills significantly increased from a median of 4 [1–7] at Intermediate-skills-test one to 10.5 [5–26] at Intermediate-skills-test three. There was no significant difference in the number of achieved skills between Intermediate-skills-test one and two and between two and three. Figure 2 shows the achieved intermediate skills per participant. Five out of ten participants showed the expected increase in number of achieved skills over the three measurements.

**Figure 3:**
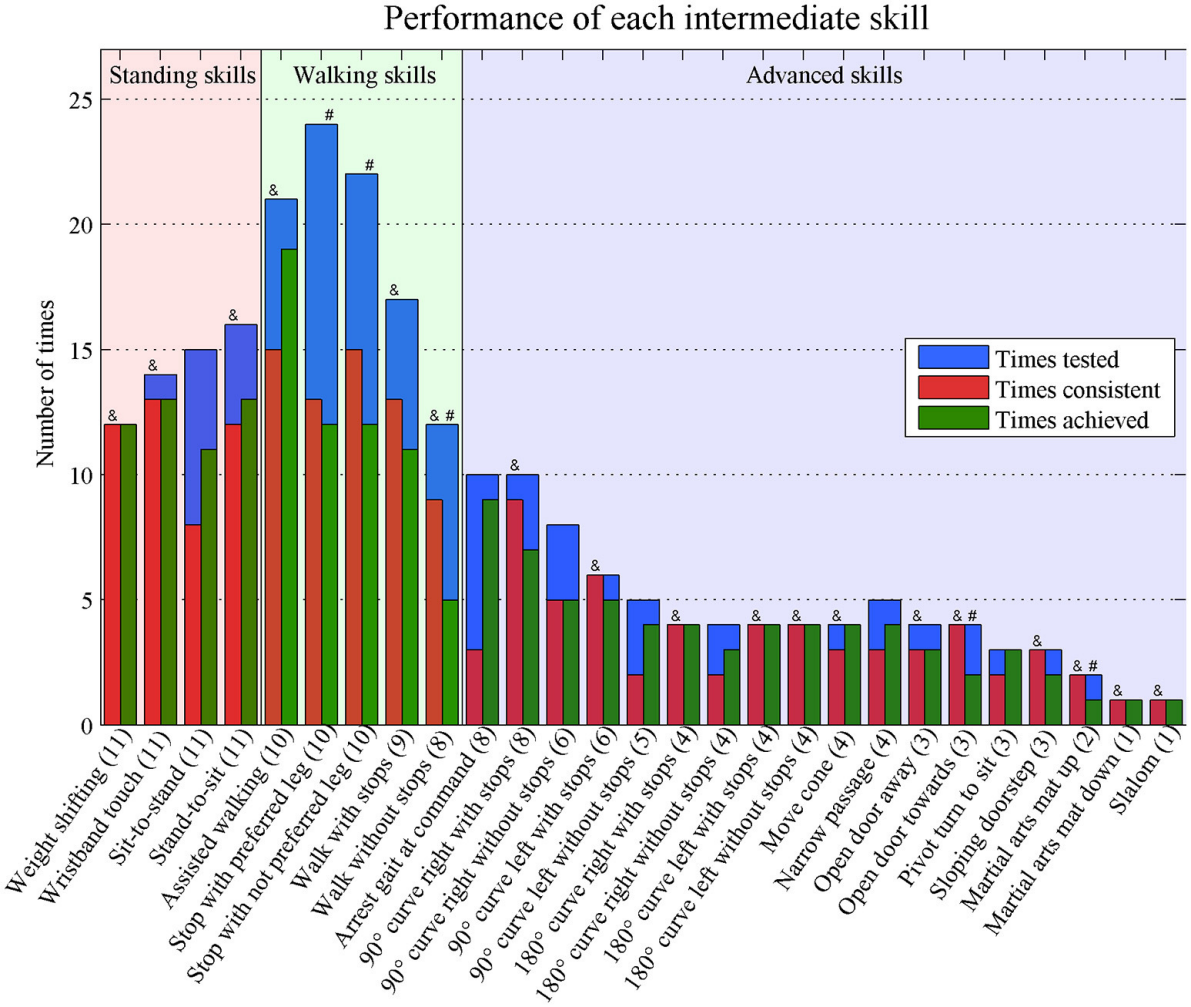
Times performed consistent and achieved of each separate intermediate skill. Numbers in brackets represent the number of tested participants. Consistent = performed the same in the first two attempts (successful-successful or failure-failure). Achieved = at least two out of three successful attempts & = >70% performed consistent # = achieved in ~50% of the times.

Detailed *post-hoc* analysis revealed that five of the intermediate skills were achieved during all measurements (see completely green bars in Figure 3). Three out of five intermediate walking skills, and two advanced skills were achieved in approximately half of the tested times, these skills are highlighted with an “#”-sign in Figure 3.

### Hierarchy of the skills

**Figure 4:**
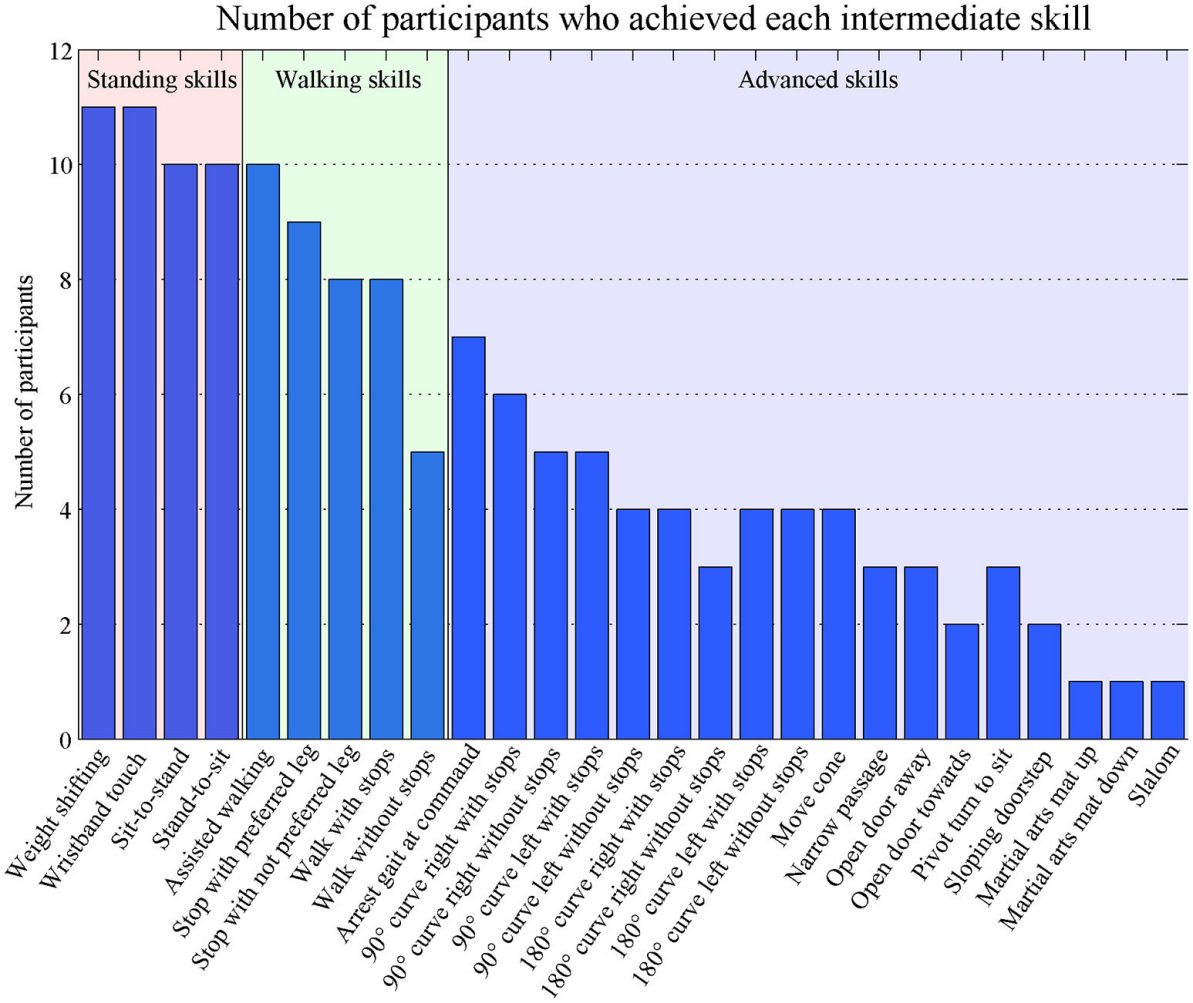
Rates of achievement of each intermediate skill.

In general, the rate of participants who achieved the intermediate skills decreased. In three skills, the “walk 10 m without stops,” “180° curve tot the right without stops,” and “open door toward”-skill, the number of participants who achieved the skills was smaller than skills later in the hierarchical order (Figure 4). The coefficient of reproducibility was 0.98 (number of scaling errors: 14, number of skills: 27 and number of observations: 31 (10 participants with three observations and one participant with one observations). The scaling errors occurred in nine different skills (Intermediate-skill 3, 4, 6, 7, 9, 10, 12, 16, and 22) and in eight out of eleven participants. Four scaling errors occurred in Intermediate-skills-test one, three in Intermediate-skills-test two, and seven in Intermediate-skills-test three.

### Achieved final skills

**Figure 5:**
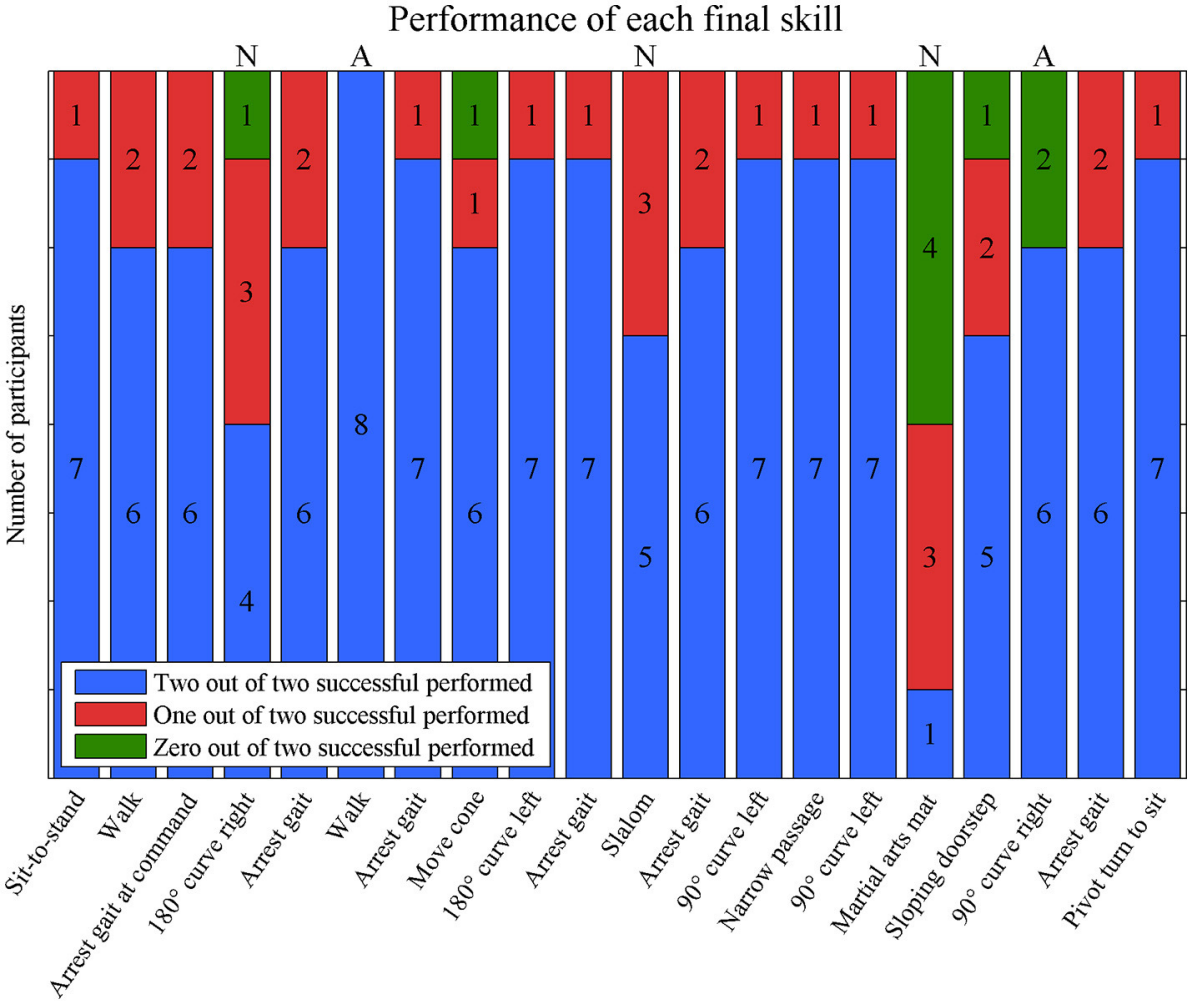
The number of consistent performances of each final skill. Green bars represent inconsistent performances in which one out of two attempt was successful performed. Blue and red bars represent consistent performances. A = all performed consistent; N = <70% performed consistent.

Eight participants were able to walk without assistance between the 18th and 23th training session and therefore met the criteria to perform the Final-skills-test. The median number of successfully performed final skills in these participants was 16.5 [13–20] and 17 [14–19] skills in the first and second time. In the Final-skills-test, 15 skills were at least one time achieved by all eight subjects (see Figure 5). The martial arts mat was not achieved by half of the participants.

The number of achieved final skills in the first and the second time were significantly correlated (*r*_τ_ = 0.75, *p* < 0.05) and not significantly different (*z* = −0.71, *p* = 0.75, effect size = −0.18). Table 3 revealed the correlation between the various skills-tests. The number of achieved skill in none of the Intermediate-skills-tests were significantly correlated with the achieved skills in any other skill-tests.

**Table 3:** Correlation (Kendall's tau) between all test moments.

	**Intermediate-skills-test 1**	**Intermediate-skills-test 2**	**Intermediate-skills-test 3**	**Final-skills-test 1**	**Final-skills-test 2**
Intermediate-skills-test 1		−0.15 (10)	0.29 (10)	0.08 (8)	0.21(8)
Intermediate-skills-test 2			0.37 (10)	0.00 (8)	−0.13(8)
Intermediate-skills-test 3				0.15 (8)	0.32(8)
Final-skills-test 1					0.75(8)^*^

*Digits in brackets represent the number of participants*.

* *Correlation is significant at the 0.05 level*.

### Consistency

Eleven participants performed in total 235 intermediate skills, of which 171 (73%) were performed the same in the first two attempts (successful-successful or failure-failure).

The number of participants who performed the skill, the number of times a skill was measured, the number of times a skill was performed consistent and the number of times a skill was achieved is shown for each intermediate skill in Figure 3. Eighteen skills were performed consistent in more than 70% of the times (highlighted with an “&”-sign in Figure 3). Of these skills, 10 skills were performed consistent during all Intermediate-skills-tests (see completely red bars in Figure 3).

Eight participant performed all twenty final skills twice resulting in a total of 160 final skills. They performed 130 (81%) final skills the same in both attempts. The median number of inconsistent performed skills per participant was 2.5 [0–9]. An overview of the consistent and inconsistent performances of each final skill is depicted in Figure 5. Most skills were performed consistently by seven (9 skills) or six (6 skills) participants. Two skills were performed consistently by all participants (highlighted with an “A”-sign in Figure 5), whereas three skills were performed inconsistent by three participants (180° curve to the right, slalom and martial arts mat) (highlighted with an “N”-sign in Figure 5).

### Complications

Eight out of twelve participants experienced device related skin damage at the feet (*n* = 3), knee (*n* = 5), thigh (*n* = 3), pelvic (*n* = 4), and/or trunk (*n* = 1) area. In four participants the skin damage resulted in at least one missed training session, which was rescheduled at the end of the training period. In case of skin damage, extra padding was added to prevent reoccurrence of the complication. As a result, most skin damage occurred in the early phase of the training program. Seven participants reported muscle or joint pain during the training program around the hands/wrists (*n* = 2), arms (*n* = 3), shoulders (*n* = 3), neck (*n* = 3), trunk (*n* = 1), and/or back (*n* = 3). None of the complications evolved into serious adverse events. During the entire study the incidence of device related errors was three times in 218 training sessions. No incontinence problems, fractures, venous-lymphatic stasis or falls were mentioned by the participants or physical therapists in the study.

## Discussion

In the present study, a framework for measuring the ability to perform basic and advanced exoskeleton skills throughout an exoskeleton training program was developed and tested. Ten participants completed the training program and were tested during the 2nd, 4th, and 6th training week. They showed an increase in the achieved intermediate skills from four at the first to 10.5 at the third Intermediate-skills-test. The rate of participants who achieved the intermediate skills decreased and the coefficient of reproducibility was 0.98. In the last training week, eight participants successfully performed 16.5 and 17 skills in the Final-skills-test. An overall consistency of 73% in the Intermediate-skills-test and 81% in the Final-skills-test was achieved.

Similar to other assistive technologies, such as prostheses and to a lesser extent wheelchairs, exoskeleton community use is preceded with a training program. Although the type and extent of the risks of exoskeletons are yet to be understood (He et al., 2017), the risks associated with exoskeleton use seems higher than with prostheses or wheelchair. The advantage of a prostheses and a wheelchair is that it can be used independently for ambulation in an early phase. Therefore, clinical tests such as the timed up and go-test (Condie et al., 2006) or the mechanical efficiency (Leving et al., 2016) can be used to evaluate the progress in performance in using the assistive technologies. In contrast, most people are not able to perform basic ambulation skills at the start of an exoskeleton training program and multiple training sessions are needed before independent ambulation is possible. Assessing performance in using assistive technologies can also be done with standardized skills-tests. For wheeled mobility several skills-test, such as the wheelchair skills-test, are available (Fliess-Douer et al., 2010). Until now there were no standardized skills-test for exoskeleton performance. Several studies marked the training session in which a skill with an exoskeleton was performed with varying levels of trainer assistance (Spungen et al., 2013; Hartigan et al., 2015; Kozlowski et al., 2015; Benson et al., 2016; Platz et al., 2016). However, in these studies the performed exoskeleton skills were kept up in a logbook and the independent achievement of skills was not tested on a regular bases. The main objective of this study was to develop a framework for measuring the ability to perform basic and advanced exoskeleton skills throughout an exoskeleton training program.

A framework to assess the progress of exoskeleton skills should consist of tests measuring achieved skills in a hierarchical order of difficulty. As a consequence participants should progress during an intensive exoskeleton training program. Although the Friedman test revealed a significant difference in the number of achieved skills between the Intermediate-skills-tests, these results should be interpreted with care because several tied ranks were observed and a small number of participants were included. Nevertheless, the number of achieved intermediate skills significantly increased from 4 at the first to 10.5 at the third Intermediate-skills-test. Furthermore, nine and seven out of ten participants had an increase in the number of achieved skills between the first and second and the second and third Intermediate-skills-test, respectively. However, such an increase in number of achieved skills doesn't automatically indicate that the skills are in a correct order of difficulty. In the current study, two measures were used to assess the hierarchy. According to the coefficient of reproducibility (0.98) the intermediate skills were in the correct order of difficulty (Guttman, 1944; Tyson and DeSouza, 2004). The rate of the number of participants achieving each skill revealed three skills (walk 10 m without stops, 180° curve to the right without stops, and open door toward-skill) that were achieved by a larger number of participants in the subsequent skill. Detailed *post-hoc* analysis revealed that only an unachieved “walk 10 m without stops”-skill was followed by achieved skills in more than two observations (five observations). Covering a distance of 10 m without stops was the last basic skills before advanced skills were tested. The advanced skills consisted of an additional task during walking such as a sudden stop, curves or passing a doorstep, but a shorter distance of ~3 m had to be covered. The length of the skill of walking a distance of 10 m increased the chance of errors and not achieving the skill. Two other studies (Spungen en Platz) also recorded the moment the 10 m walking skill was performed without assistance, four (Spungen et al., 2013) and one (Platz et al., 2016) out of seven participants were able to perform the skill independent within 24 training sessions. Indicating the difficulty of walking 10 m without assistance. In the current study each test session was scheduled in advance within a training session and an extra person was present during the skills-test. As a result, most participants were more stressed during the skills-test than during other training sessions. An increased stress level could evoke more spasticity (Fleuren et al., 2009) causing involuntary stops, which particularly influenced the achievement of the “10 m walking skill without stops”-skill. Because walking without stops is crucial in performing most advanced skills, we prefer to keep the proposed order of the skills. However, for future research we would advise to change the order of the Intermediate-skills for the “walking curves” according to the preference of the patient.

In addition to assess the progression, the framework had to discriminate across participants. In all Intermediate-skills-tests, differences between participants were apparent. After 2 weeks of exoskeleton training participants were only able to perform basic skills, but varied between standing and walking skills. This was in line with the findings of Spungen et al. (2013). In the current study, all participants were able to perform all intermediate standing skills without assistance after 4 and 6 weeks of training, but differed in walking and advanced skills. In addition to differences across participants at the Intermediate-skills-tests, participants showed various learning curves. The low correlation between the three Intermediate-skills-tests (*r*_τ_ between −0.15 and 0.37) supports the various learning process across participants. In conclusion, the framework proposed in this study measured the progress in the ability to perform basic and advanced exoskeleton skills, had the skills in a hierarchical order of difficulty and could discriminate across participants.

A second important prerequisite of the framework is that the tested skills were reliable. An overall consistency of 73% in the Intermediate-skills-test and 81% in the Final-skills-test was achieved. Detailed analysis revealed several skills that had a consistency of <70% (see Figures 3, 5). Remarkable was a lower consistency for the same skills in the Intermediate-skill-test compared to the Final-skill-test or vice versa. A consistency below 70% in the Intermediate-skill-test whereas a consistency above 70% in the Final-skill-test was met for the skills: sit-to-stand, stopping with the preferred or not preferred leg, arresting gait at command, passing a narrow passage, and pivot-turn to sit. The lower consistency of the intermediate skills were possibly due to the learning process. During the Intermediate-skills-test participants had to perform skills they practiced only once or twice without assistance. As a result, the skill was sometimes successfully performed by chance instead of competence and therefore participants were unable to perform the skill consistent. Therefore, we recommend that a skill should be performed at least two times when tested. The skills 180° curve to the right, slalom and martial arts mat had a low consistency in the Final-skills-test whereas a high consistency was obtained in the Intermediate-skills-test. These skills were performed by only a minority of the participants during the Intermediate-skills-tests indicating that most participants were only in the last training sessions at a level that they could practice these advanced skills. Nevertheless, the majority of the tested skills were performed consistent in the Intermediate- and Final-skills-test. Therefore, considering a skill achieved after two out of three successful attempts seems a good assumption to evaluate the skill-level.

In order to achieve exoskeleton skills, participants received multiple training sessions per week over an 8 week period. Such an intensive training program yields the potential of complications such as bruises and other skin damage. Most previous studies indicated that in hospital training with an exoskeleton was safe (Zeilig et al., 2012; Esquenazi, 2013; Kolakowsky-Hayner et al., 2013; Kubota et al., 2013; Spungen et al., 2013; Miller et al., 2016). Although other studies disclosed mild to moderate skin damages in half of the participants (five out of ten) (Benson et al., 2016) (four out of seven) (Platz et al., 2016), the intensity (session per week) and duration of the training period in this study was similar to most previous studies (Spungen et al., 2013; Benson et al., 2016; Miller et al., 2016; Platz et al., 2016). In the current study, eight out of twelve participants experienced skin damage during the training program. In four cases this skin damage resulted in at least one missed training. Whereas all skin damages reported in the study of Benson and none of the skin damages reported by Platz led to discontinuation of the training (Benson et al., 2016; Platz et al., 2016). Because of extra padding in the early phase of the training program, skin damage rarely occurred in the later phase. In addition, during the whole training program special care was taken to the correct joint alignment. Therefore, none of the patients had to reduce the training intensity in the later phase of the training program. Moreover, it suggests that special attention to joint alignment and padding during the training reduces the risk of skin damage.

In the current study, the Rewalk exoskeleton was used. In the current study, the ReWalk exoskeleton was used. Nowadays, there are multiple exoskeletons available on the market, which have their specific interaction with and control of the exoskeleton. As a consequence the hierarchical order of the skills in the framework might be slightly different between exoskeletons. The main difference between the current available exoskeletons for home and community is the control of initiation of gait, arresting of gait, and involuntary stops. For example, to initiate gait, the ReWalk and Ekso require a forward and lateral shift of the trunk, the Indego exoskeleton requires a forward trunk excursion, and the Rex exoskeleton does not require any trunk movement (Bryce et al., 2015). Despite the differences in interaction with and control of the exoskeleton, all skills proposed in the current study are relevant and applicable to the current available exoskeletons. Standing and walking skills are presumed to be achieved before users can perform additional advanced skills, which are mostly performed during walking. The first eight advanced skills, require less interaction with the environment. Within these skills a distinction was made in the fluency of the performance (with or without stops). Therefore, we expect that the hierarchical order of the first 18 intermediate skills can be applied to other exoskeletons. The hierarchical order of the last nine advanced skills might be slightly different across exoskeletons due to the difference in interaction with and control of the exoskeleton. However, achieving one of these skills indicates a highly advanced exoskeleton skill level.

The skills-tests proposed in this framework were based on independent performance of exoskeleton skills, but did not take the quality of the performance into consideration. For future research, the quality of how a skill is performed might be of interest in addition to if it is possible to perform a skill independent. Moreover, all exoskeleton skills in this study were assessed in a clinical setting and it remains unknown which skills are relevant and which skill-level is necessary for safe community use. Therefore, future research should focus on community use of an exoskeleton and its relation to the skill-level during the training period measured with the proposed framework.

## Conclusion

The framework proposed in this study measured the progress in performing basic and advanced exoskeleton skills during a training program. The hierarchical ordered skills-test could discriminate across participants' skill-level. The overall consistency of the performed exoskeleton skills was considered acceptable.

## Disclosure

The exoskeleton training of the physiotherapists by ReWalk Robotics was given before the start of the study and ReWalk Robotics does not have any influence on the entire study.

## Author contributions

All authors were involved in the design of the study. RvD, HR, and NK conceived and planned the experiments. RvD performed the data collection during the experiments. RvD and NK processed the experimental data, performed the analysis, drafted the manuscript and designed the figures. IvN, HvdM, and NK supervised the project. All authors discussed the results and contributed to the final manuscript.

### Conflict of interest statement

The authors declare that the research was conducted in the absence of any commercial or financial relationships that could be construed as a potential conflict of interest.
