# Supplementary material for: A Framework for Measuring the Progress in Exoskeleton Skills in People with Complete Spinal Cord Injury
Source: Front Neurosci. 2017 Dec 12;11:699. doi: 10.3389/fnins.2017.00699 (PMC5732998; doi:10.3389/fnins.2017.00699)
Supplement: Supplementary file 1 [file Table1.docx]

**Supplementary material**

Table 1. Intermediate-skills-test

| **Fill out** | **Intermediate-skill** | **Task*** | **Additional requirements** |
| --- | --- | --- | --- |
| Successful? 1*.* 1 YES NO  2 YES NO  3 YES NO | **Weight shifting in stance** | Shift the weight to the front (while moving the crutches in front of you), to the back (move the crutches to the back), to the left (right foot is cleared from the floor) and to the right (left foot is cleared from the floor). | All four positions have to maintained for at least two seconds. |
| Successful? 2*.* 1 YES NO  2 YES NO  3 YES NO | **Wristband touch in stance** | Touch your left wrist with your right hand. Put both crutches back on the floor and then touch your right wrist with your left hand. | Perform each wristband touch within ten seconds. |
| Successful? 3*.* 1 YES NO  2 YES NO  3 YES NO | **Sit-to-stand** | Perform sit-to-stand.  *The sit-to-stand and stand-to-sit skill are measured alternately.* | The crutches may be placed in the right place after confirmation of the standing mode. |
| Successful? 4*.* 1 YES NO  2 YES NO  3 YES NO | **Stand-to-sit** | Perform stand-to-sit. | The crutches may be placed in the right place after confirmation of the standing mode. |
| Successful? 5*.* 1 YES NO  2 YES NO  3 YES NO | **Assisted walking** | Walk 10m straight with assistance of the exoskeleton trainer. | The maximum allowed number of stops is two. |
| Successful? 6*.* 1 YES NO  2 YES NO  3 YES NO | **Arrest gait with preferred leg** | Start walking and stop as close as possible to a pre-specified line with the preferred leg. The pre-specified line is approximately 3m away from the starting point. | Stop in a 50 cm range of the pre-specified line. |

| **Fill out** | **Intermediate skill** | **Task*** | **Additional requirements** |
| --- | --- | --- | --- |
| Successful? 7. 1 YES NO  2 YES NO  3 YES NO | **Arrest gait with not preferred leg** | Start walking and stop as close as possible to a pre-specified line with the not preferred leg. The pre-specified line is approximately 3m away from the starting point. | Stop in a 50 cm range of the pre-specified line. |
| Fill out the 8. number of stops  1……………  2……………  3……………    9. | **Walk 10m with stops**  Successful?  1 YES NO  2 YES NO  3 YES NO | Walk 10m straight without assistance of the exoskeleton trainer.  *The walk 10m with stops and without stops skills are measured simultaneously.* | The maximum allowed number of stops is two within the 10m. |
|  | **Walk 10m without stops**  Successful?  1 YES NO  2 YES NO  3 YES NO | Walk 10m straight without assistance of the exoskeleton trainer. | There are no stops allowed within the 10m. |
| Successful? 10*.* 1 YES NO  2 YES NO  3 YES NO | **Arrest gait at**  **command** | While walking straight ahead stop as fast as possible when the ‘Stop’ command is given.  The stop command is given at either the left or right heel strike. | Stop within one stride. |
| Fill out the 11. number of stops  1……………  2……………  3……………    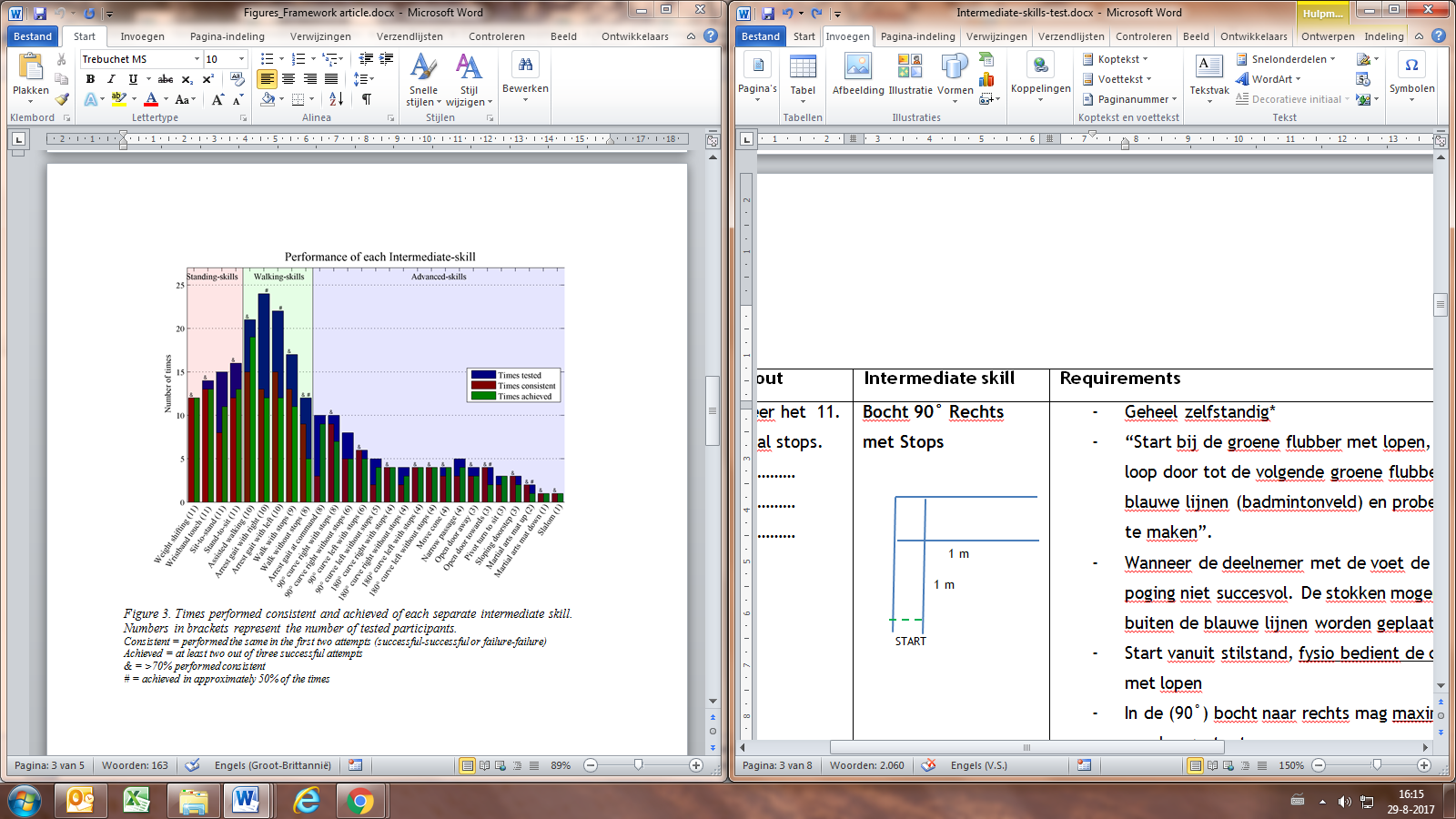 12. | **90˚curve right with stops**  Successful?  1 YES NO  2 YES NO  3 YES NO | Walk a 90˚curve to the right within the lines of the badminton field.  *The walk 90˚curve with stops and without stops skills are measured simultaneously.* | The maximum allowed number of stops is one.  The feet should be within the lines of the badminton field, the crutches are allowed outside the lines. |
|  | **90˚curve right without stops**  Successful?  1 YES NO  2 YES NO  3 YES NO | Walk a 90˚curve to the right. | There are no stops allowed. The feet should be within the lines of the badminton field, the crutches are allowed outside the lines. |

| **Fill out** | **Intermediate skill** | **Task*** | **Additional requirements** |
| --- | --- | --- | --- |
| Fill out the 13. number of stops  1……………  2……………  3……………  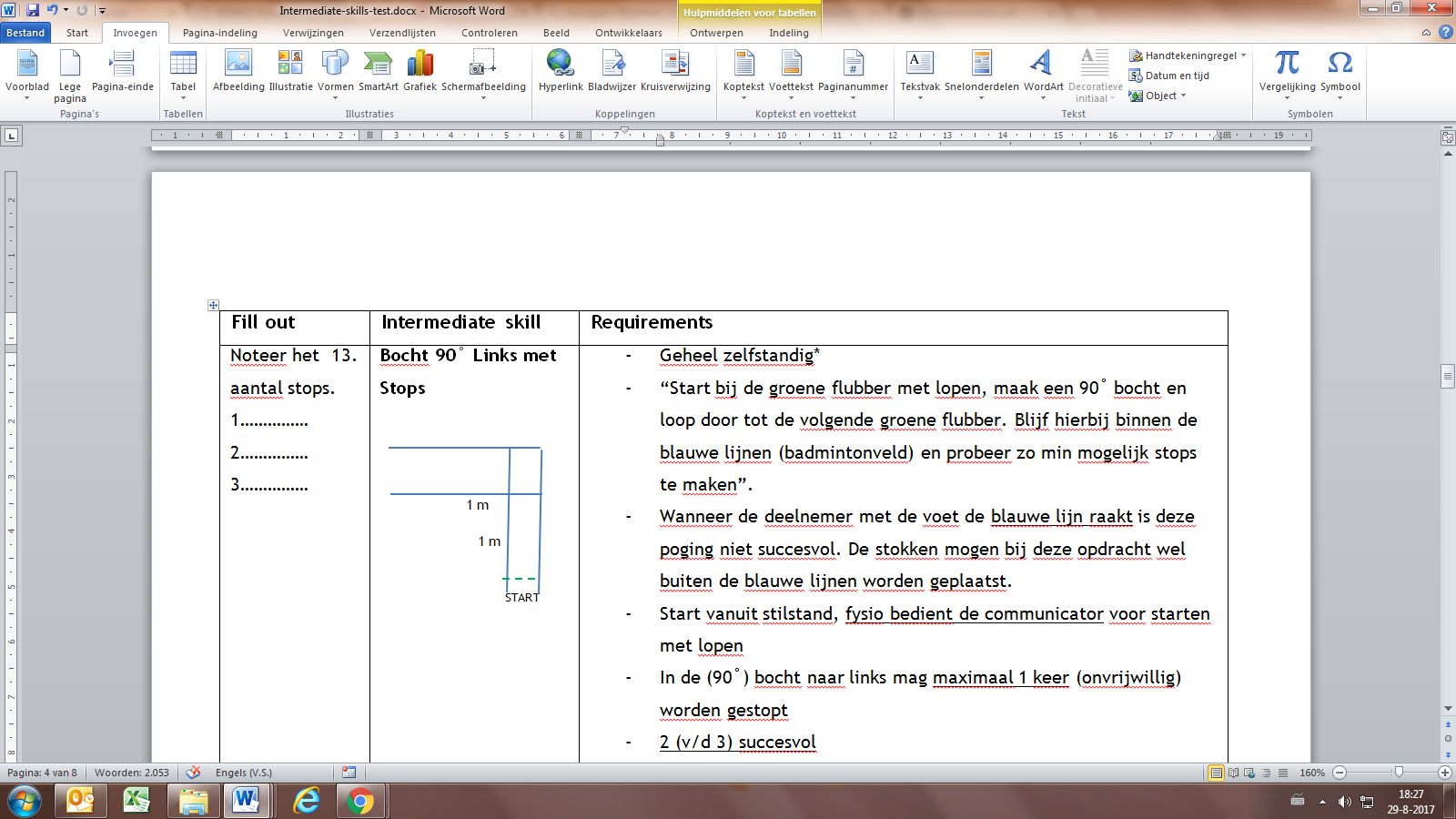  14. | **90˚curve left with stops**  Successful?  1 YES NO  2 YES NO  3 YES NO | Walk a 90˚curve to the left within the lines of the badminton field.  *The walk 90˚curve with stops and without stops skills are measured simultaneously.* | The maximum allowed number of stops is one.  The feet should be within the lines of the badminton field, the crutches are allowed outside the lines. |
|  | **90˚curve left without stops**  Successful?  1 YES NO  2 YES NO  3 YES NO | Walk a 90˚curve to the left. | There are no stops allowed. The feet should be within the lines of the badminton field, the crutches are allowed outside the lines. |
| Fill out the 15. number of stops  1……………  2……………  3……………    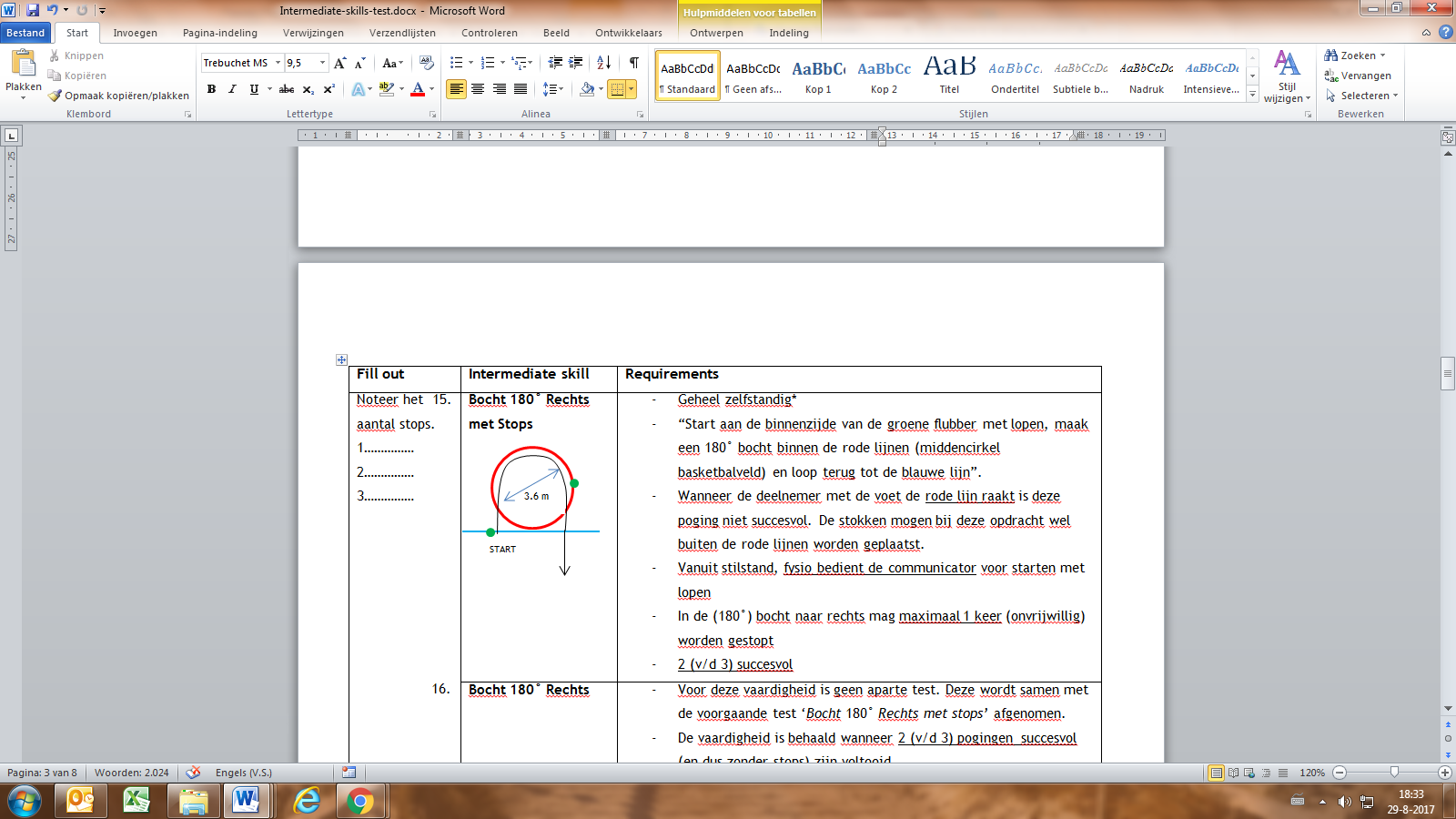 16. | **180˚curve right with stops**  Successful?  1 YES NO  2 YES NO  3 YES NO | Walk a 180˚curve to the right within the middle circle of the basketball court.  *The walk 180˚curve with stops and without stops skills are measured simultaneously.* | The maximum allowed number of stops is one.  The feet should be within the middle circle of the basketball court, the crutches are allowed outside the circle. |
|  | **180˚curve right without stops**  Successful?  1 YES NO  2 YES NO  3 YES NO | Walk a 180˚curve to the right. | There are no stops allowed. The feet should be within the middle circle of the basketball court, the crutches are allowed outside the circle. |
| Fill out the 17. number of stops  1……………  2……………  3……………    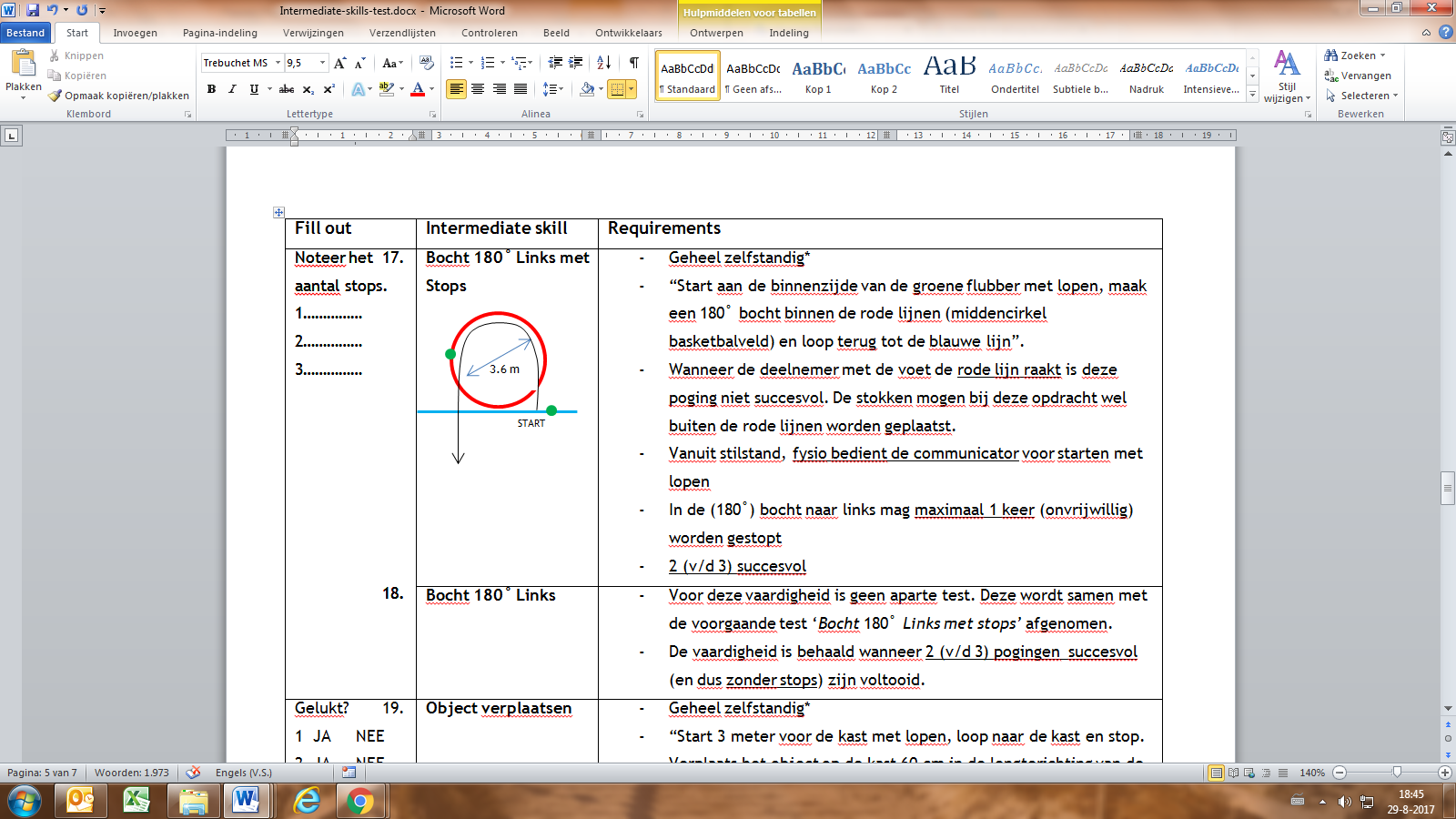 18. | **180˚curve left with stops**  Successful?  1 YES NO  2 YES NO  3 YES NO | Walk a 180˚curve to the left within the middle circle of the basketball court.  *The walk 180˚curve with stops and without stops skills are measured simultaneously.* | The maximum allowed number of stops is one.  The feet should be within the middle circle of the basketball court, the crutches are allowed outside the circle. |
|  | **180˚curve left without stops**  Successful?  1 YES NO  2 YES NO  3 YES NO | Walk a 180˚curve to the left. | There are no stops allowed. The feet should be within the middle circle of the basketball court, the crutches are allowed outside the circle. |

| **Fill out** | **Intermediate skill** | **Task*** | **Additional requirements** |
| --- | --- | --- | --- |
| Successful? 19*.* 1 YES NO  2 YES NO  3 YES NO | 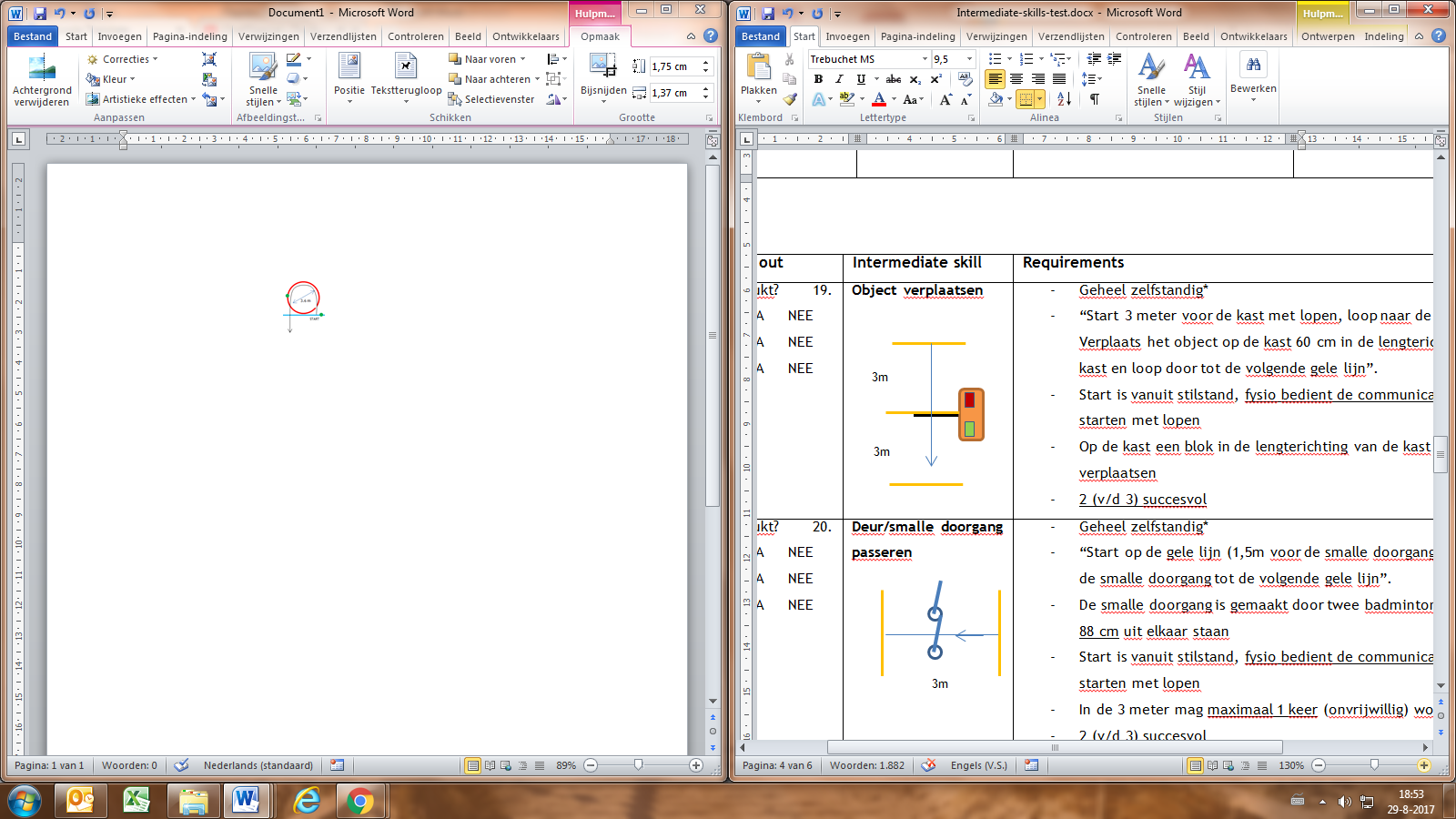**Move cone** | Walk straight and arrest gait near the vaulting box. Move the cone 60 cm in the longitudinal direction of the vaulting box and continue walking. | The vaulting box may be used for balance support while moving the cone. |
| Successful? 20*.* 1 YES NO  2 YES NO  3 YES NO | 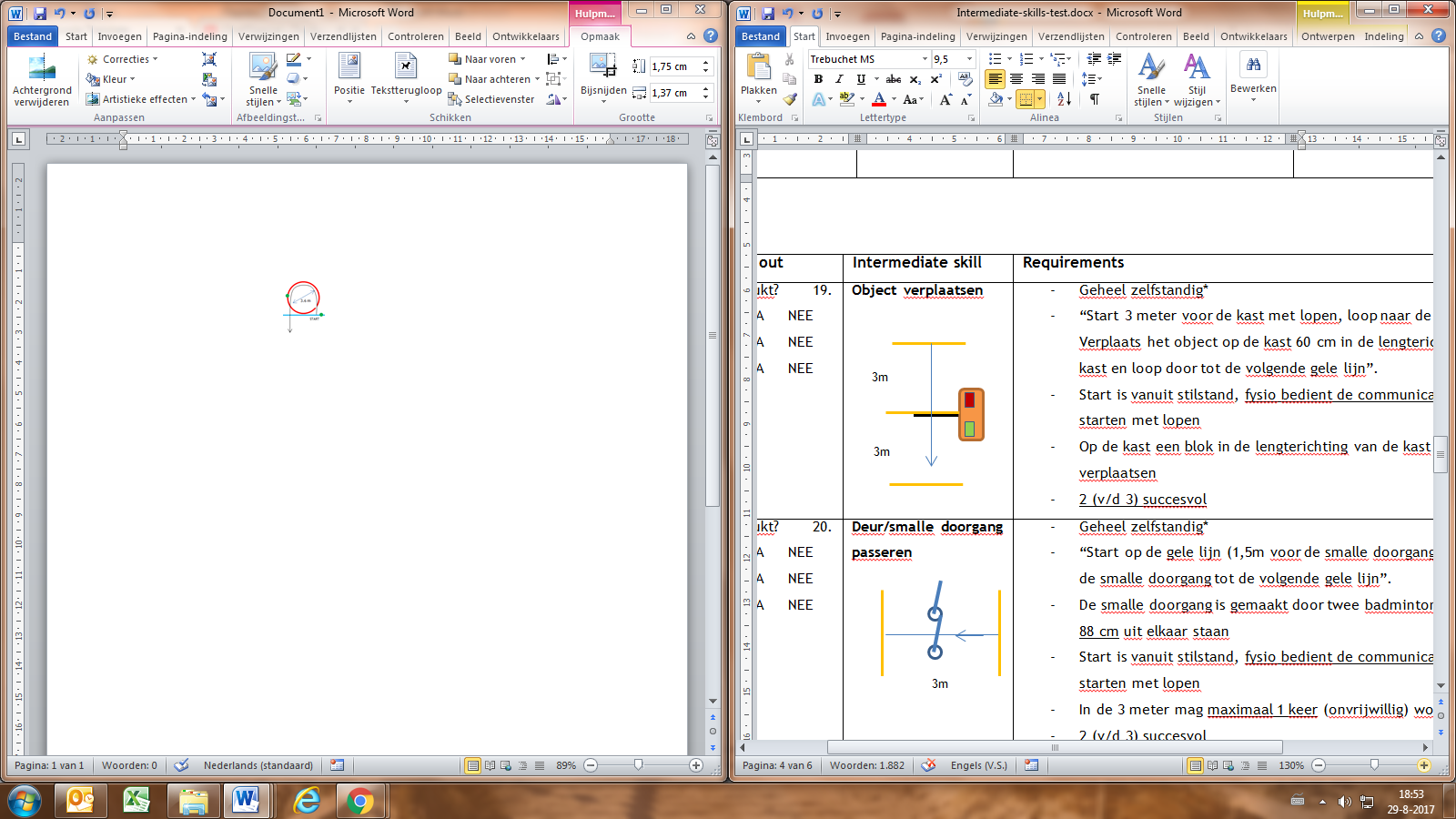**Narrow passage** | Walk towards the narrow passage made out of two badminton poles and pass the passage (width 0.8m) | The maximum allowed number of stops is one. |
| Successful? 21*.* 1 YES NO  2 YES NO  3 YES NO | **Open door away** | Arrest gait nearby a door (width 0.8m), open the door away from you and enter.  *The open door away and towards skill are measured alternately.* | The maximum allowed number of stops is one. |
| Successful? 22*.* 1 YES NO  2 YES NO  3 YES NO | **Open door towards** | Arrest gait nearby a door (width 0.8m), open the door towards you and enter. | The maximum allowed number of stops is one. |
| Successful? 23*.* 1 YES NO  2 YES NO  3 YES NO | **Pivot turn to sit** | Arrest gait near a chair (height 0.5m) and pivot turn to sit down |  |
| Successful? 24*.* 1 YES NO  2 YES NO  3 YES NO | **Pass a sloping doorstep** | Pass an upward and downward sloping doorstep (angle up 11.3˚and down 16.7˚, height 0.03m) | The maximum allowed number of stops is one. |
| Successful? 25*.* 1 YES NO  2 YES NO  3 YES NO | **Walk up a martial arts mat** | Walk up a martial arts mat (height 0.04m)  *The up and down a martial arts mat skill are measured alternately.* | The maximum allowed number of stops is one. |
| Successful? 26*.* 1 YES NO  2 YES NO  3 YES NO | **Walk down a martial arts mat** | Walk down a martial arts mat (height 0.04m) | The maximum allowed number of stops is one. |
| Successful? 27*.* 1 YES NO  2 YES NO  3 YES NO | 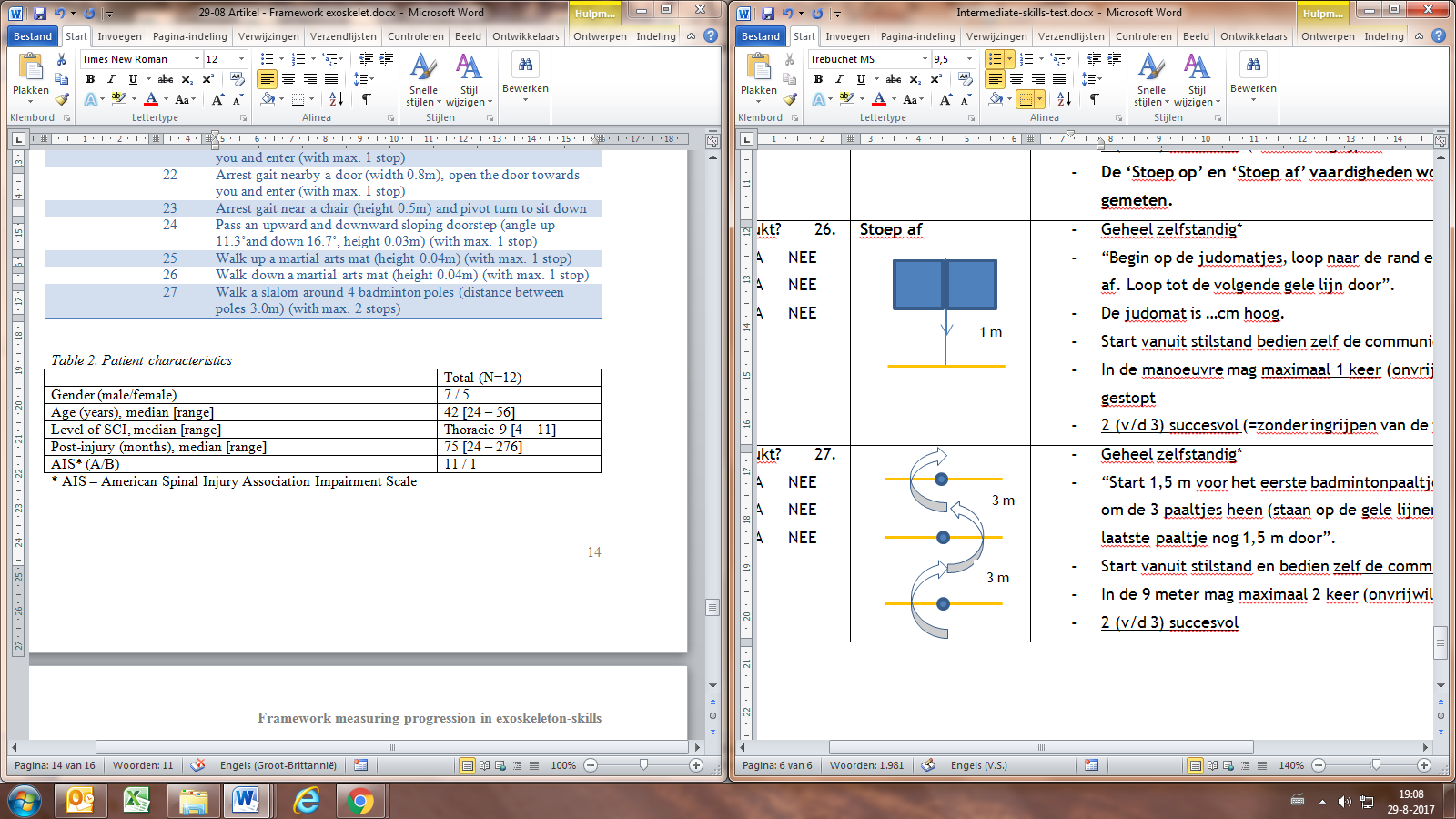**Walk a slalom** | Walk a slalom around 4 badminton poles (distance between poles 3.0m) | The maximum allowed number of stops is two. |
